# Anatomy and size of *Megateuthis*, the largest belemnite

**DOI:** 10.1186/s13358-024-00320-x

**Published:** 2024-05-30

**Authors:** Christian Klug, Günter Schweigert, René Hoffmann, Dirk Fuchs, Alexander Pohle, Robert Weis, Kenneth De Baets

**Affiliations:** 1 Paläontologisches Institut und Museum, Universität Zürich, Karl-Schmid-Strasse 4, 8006 Zurich, Switzerland; 2 Staatliches Museum für Naturkunde, Rosenstein 1, 70191 Stuttgart, Germany; 3 Institute of Geology, Mineralogy, & Geophysics, Ruhr-Universität Bochum, 44801 Bochum, Germany; 4 SNSB-Bayerische Staatssammlung für Paläontologie und Geologie, Richard-Wagner-Straße 10, 80333 Munich, Germany; 5 Section Paléontologie 25, Musée National d’histoire Naturelle, Rue Münster, L-2160 Luxembourg, Luxembourg; 6 Institute of Evolutionary Biology, Faculty of Biology, University of Warsaw, Ul. Żwirki I Wigury 101, 02-089 Warsaw, Poland

**Keywords:** Cephalopoda, Belemnitida, Bajocian, Anatomy, Gigantism, Taphonomy

## Abstract

Belemnite rostra are very abundant in Mesozoic marine deposits in many regions. Despite this abundance, soft-tissue specimens of belemnites informing about anatomy and proportions of these coleoid cephalopods are extremely rare and limited to a few moderately large genera like *Passaloteuthis* and *Hibolithes*. For all other genera, we can make inferences on their body proportions and body as well as mantle length by extrapolating from complete material. We collected data of the proportions of the hard parts of some Jurassic belemnites in order to learn about shared characteristics in their gross anatomy. This knowledge is then applied to the Bajocian genus *Megateuthis*, which is the largest known belemnite genus worldwide. Our results provide simple ratios that can be used to estimate belemnite body size, where only the rostrum is known.

## Introduction

**Fig. 1:**
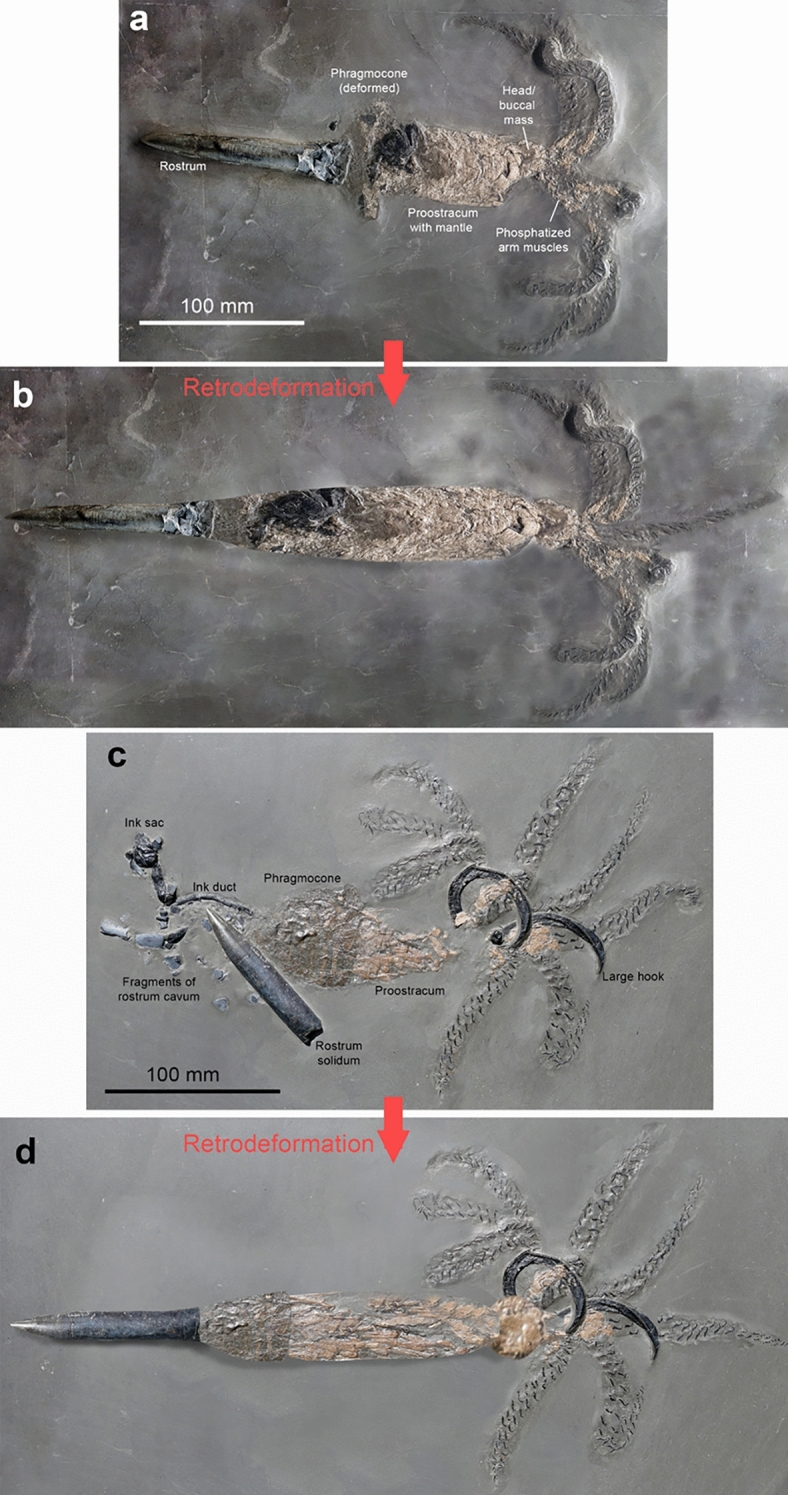
Complete specimens of *Passaloteuthis bisulcata* from the early Toarcian, Tenuicostatum Zone, Semicelatum Subzone, Ohmden, Germany. Both specimens were deformed, perhaps by predation or by compaction of oblique embedding (sinking rostrum first). Note that no scale bars were added in b and d because these are distorted versions of **a** and **c**. **a** Perhaps a female (**a**) because of the ten similar arms from the Museum Hauff (Holzmaden). **c** Perhaps a male (**b**) because of the large hooks from the Staatliches Museum für Naturkunde in Stuttgart, SMNS 70559). **b** and **d** show the same specimens retro-deformed using PhotoShop with the approximate longitudinal proportions

Belemnites preserving parts other than the mostly calcitic rostrum and the aragonitic phragmocone are exceedingly rare (Hoffmann & Stevens, 2020; Schlegelmilch, 1998; Weis & Mariotti, 2007). They are so rare that fifty years ago, a forged specimen, a ‘chimera’, was produced using an actual belemnite rostrum, which was skilfully glued to a belemnotheutid phragmocone, then taken for real, and published (Seilacher & Wiesenauer, 1978; Wiesenauer, 1976). Soon thereafter, true complete belemnites were discovered, and the forged specimens identified as such (Riegraf & Reitner, 1979). In the meantime, several specimens preserving not only rostrum and phragmocone but also the hooklet-bearing arms, the poorly mineralized proostracum and actual phosphatized soft parts (Fig. 1) were found in the Toarcian of Holzmaden as well as the Kimmeridgian of Nusplingen and the Tithonian of Franconia in Germany (Reitner & Urlichs, 1983; Riegraf & Hauff, 1983; Urlichs et al., 1994; Schlegelmilch, 1998; Fuchs, 2006; Klug et. al. 2010, 2021; Heyng, 2019). No such fossils are known from comparable strata in, e.g., France or Great Britain, which seems to be a taphonomic artefact, i.e. in oxygenated settings, scavengers would have eaten up most of the belemnite soft tissue. Other factors (such as anatomy and physiology) might have generally exacerbated reduced soft-tissue fidelity in this group (Clements et al., 2017).

The most abundant preserved fossil remains of belemnites are the calcitic rostra. They are regionally so common that they even form mass accumulations often called ‘battlefields’ (Doyle & Macdonald, 1993; Rita et al., 2018). Irrespective of the incomplete knowledge of belemnite anatomy, their regionally extremely abundant calcitic rostra reflect their key role in Jurassic and Cretaceous marine ecosystems (Hoffmann & Stevens, 2020). Similar to ammonoids, they likely produced large amounts of small, planktonic offspring (Arkhipkin & Laptikhovsky, 2012; De Baets et al., 2012; Fuchs et al., 2020; Tajika et al., 2018) and had a higher metabolism than nautilids (Tajika et al., 2020, 2023), which at least contributed if not directly caused their demise during the Late Cretaceous. In order to better understand their ecological roles, reconstructing their anatomy, proportions of soft and hard parts, and body size are relevant to place them adequately in the food webs. Scarce direct evidence for belemnoid diet has been provided in recent years by Klaschka (2018), Jenny et al. (2019), Hart et al. (2020) and Klug et al. (2021). In these cases, belemnoids have fishes and crustacean remains in their arm crowns (for a younger record of a coleoid catching a fish see Mironenko et al., 2021). In the upper Kimmeridgian Plattenkalk (platy limestone) of Nusplingen, an accumulation of small bitten aptychi (calcitic lower jaw element of ammonites) was associated with a belemnite rostrum and fossilized ink and is interpreted as its stomach content (Schweigert, 2018).

In recent decades, belemnites have gained importance because of their use in isotope geochemistry (e.g., Dera et al., 2009; Hoffmann & Stevens, 2020; Mutterlose et al., 2010; Stevens et al., 2014 and references therein), to study diversity and size changes (De Baets et al., 2021; Neige et al., 2021; Rita et al., 2019) as well as disparity analyses (Dera et al., 2016; Nätscher et al., 2021) across extinction events. Ippolitov et al. (2018), Hoffmann and Stevens (2020) and Stevens et al. (2022) demonstrated how different materials such as calcite, aragonite, and organic components are distributed in the rostrum. Novel studies have also examined their phylogeny in greater detail (Stevens et al., 2023), placing *Megateuthis* firmly within Belemnitida.

*Megateuthis* is an iconic genus since its rostrum reaches the by far greatest sizes of all known belemnoids. Most figured rostra measure less than 70 cm in length (e.g., Klug et al., 2018; Schlegelmilch, 1998; Weis & Mariotti, 2007). Schlegelmilch (1998: p. 75) reported rostra of up to 80 cm length and phragmocones of up to 20 cm diameter, although without illustrating them. There are rostra in private collections measuring around 110 cm, but these are probably composites of several specimens. The largest rostra we could find are around 70 cm in length and the largest phragmocone is about 16 cm wide.

Here, we use published and museum specimens of *Megateuthis* to reconstruct the size and proportions of its soft parts. Using body proportions of the few completely known belemnoids (belemnitids and belemnotheutids), we then provide a series of estimates for the body size of *Megateuthis*. Finally, we discuss the ecological role of the genus in the light of a possible Bajocian gigantism.

## Material and methods

**Fig. 2:**
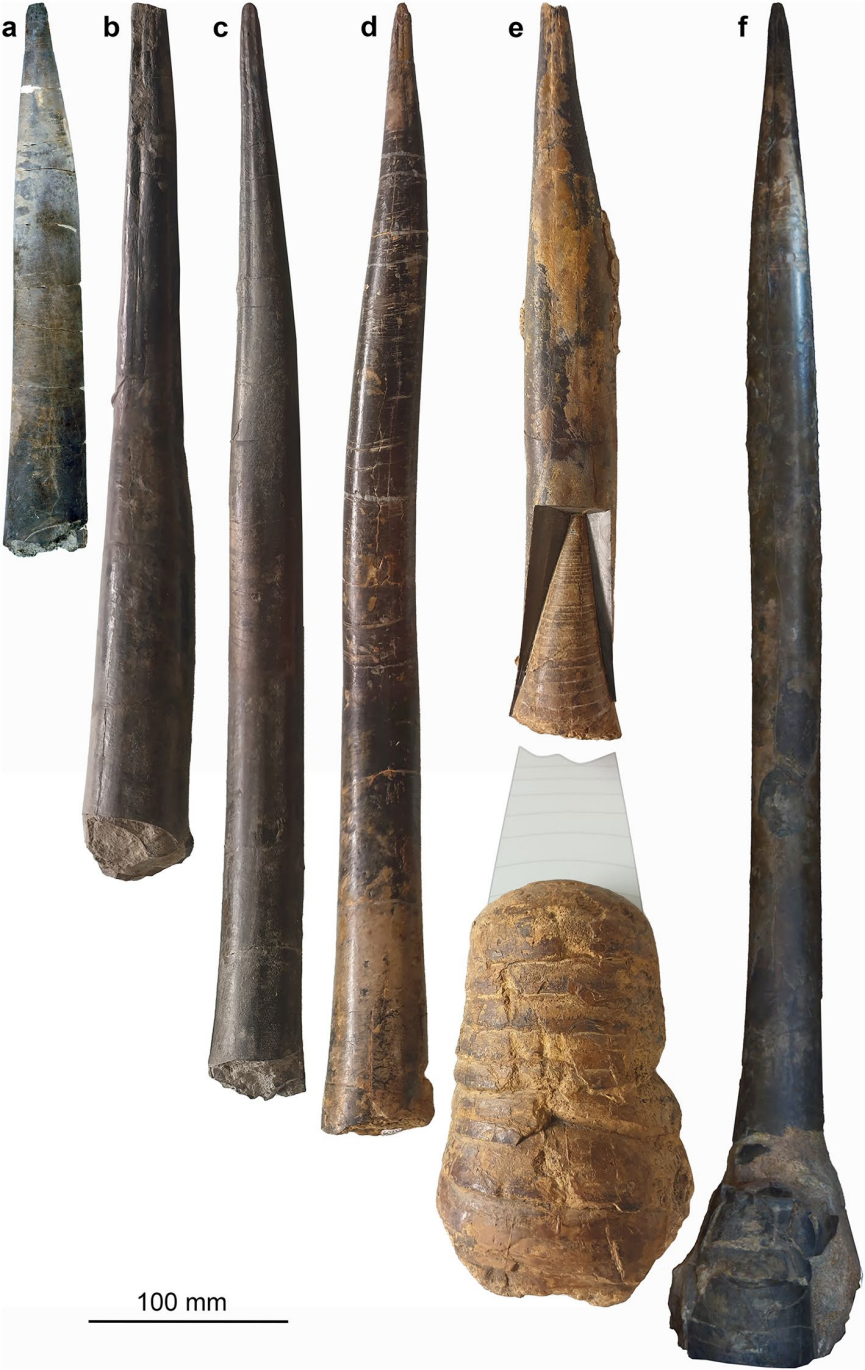
Rostra (lateral views) of *Megateuthis* from the Bajocian (Middle Jurassic) of Switzerland (**a**) and Germany (**b** to **f**). **a** *M. suevica*, PIMUZ 39843, Auenstein, Aargau. **b** *M. suevica*, SMNS 23169, Eningen unter Achalm. **c** *M. elliptica*, SMNS 61019, Eningen unter Achalm. **d** *M. elliptica*, SMNS 60752, Bopfingen-Oberdorf. **e** *M. suevica*, SMNS 61017 and 61018 (phragmocone), Bopfingen-Oberdorf. **f** *M. elliptica*, Garantiana layer, Winnberg, private collection of Paul Winkler

**Fig. 3:**
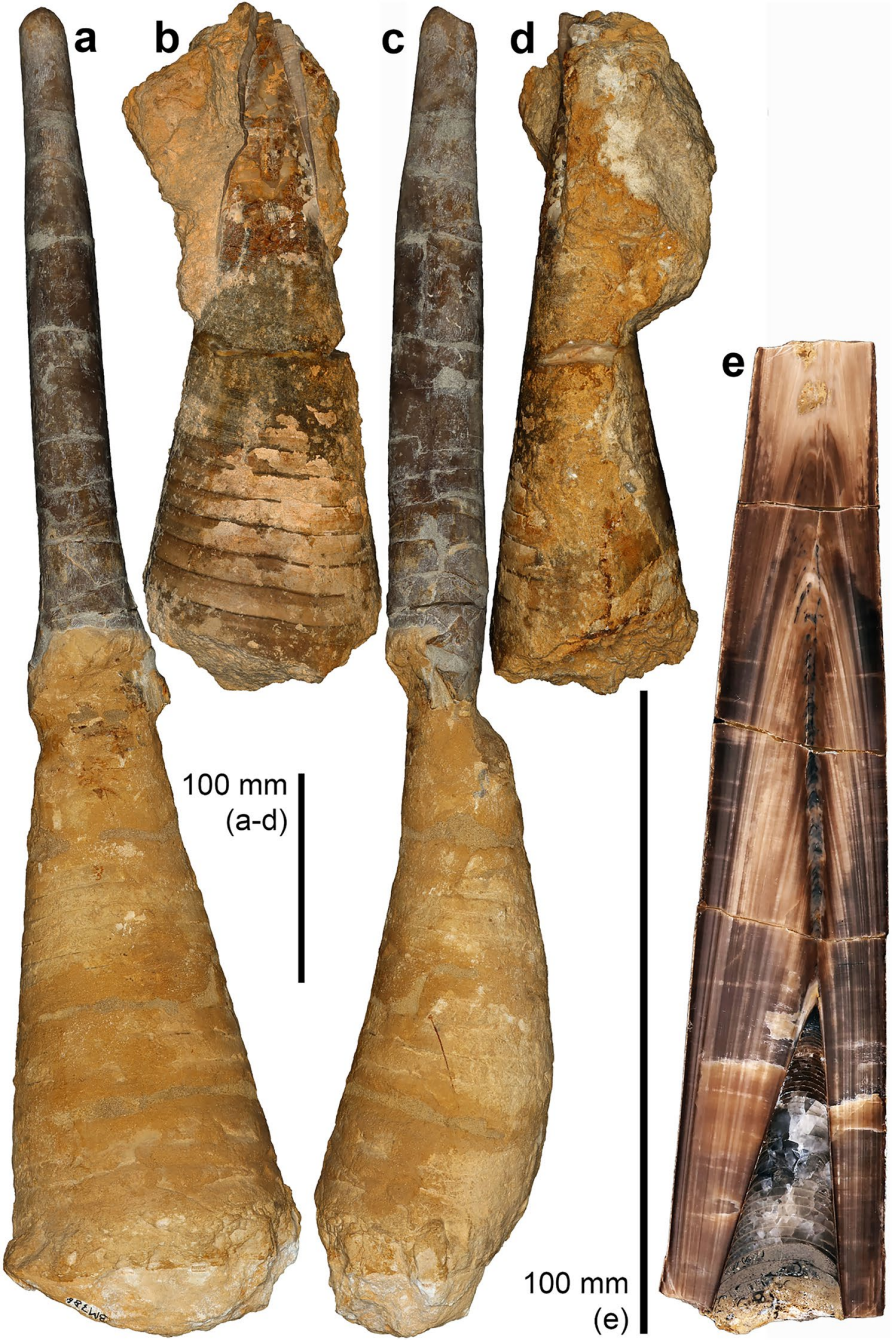
*Megateuthis* from the Middle Jurassic of Rumelange Luxembourg. **a**, **c**, nearly complete rostrum and phragmocone of *M. suevica*, MNHNL BM786. **b**, **d** phragmocone of *M*. sp., MNHNL BM648. **e** longitudinally cut rostrum with apical part of the phragmocone of *M*. sp., MNHNL BM350

*Megateuthis* is a rather common and geographically widespread belemnitid. While the members of the family Megateuthidae range from the Toarcian to the Kimmeridgian (Hoffmann & Stevens, 2020; Ippolitov et al., 2017), the genus itself (including the former subgenus *Mesoteuthis*; see Doyle, 1990) is known so far from the upper Toarcian to the Bajocian of the northern hemisphere and comprises numerous species. The Bajocian taxa *M. elliptica* and *M. suevica* (*M. gigantea* is a junior synonym according to Riegraf, 2001) are iconic for the giant dimensions of their rostra (over 30 cm). *Megateuthis* is well known from the Bajocian of France, Germany (Fig. 2), Greenland, Great Britain, Italy, Luxembourg (Fig. 3), Novaya Zemlya (sensu Doguzhaeva et al., 2002), and Switzerland (Fig. 2). *Megateuthis* belongs to an exclusively Jurassic clade of belemnites, the Belemnitina, which also includes the Passaloteuthidae (Stevens et al., 2023).

**Fig. 4:**
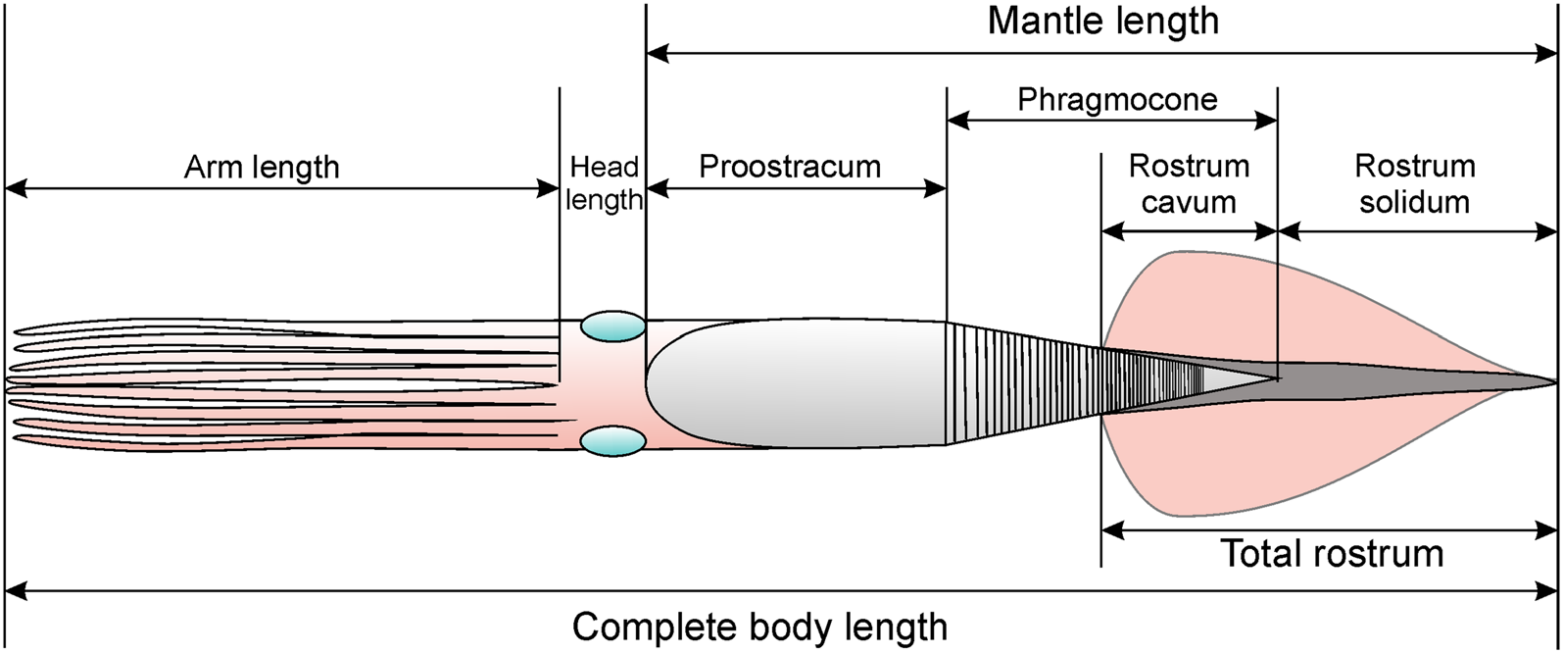
Measurements used herein, partially following Schlegelmilch (1998)

The basis of our size estimates are specimens housed in various collections such as the collection of the Department of Palaeontology of the University of Zurich (PIMUZ numbers), the Musée national d’histoire naturelle, Luxembourg (MNHNL numbers), and the Staatliches Museum für Naturkunde in Stuttgart (SMNS numbers). We added published data where we considered the material and measurements as trustworthy because appropriate photos with scale were provided.

To determine the range of body proportions of belemnoids, we used the few specimens that have been published with hard and soft parts (see Tables 1, 2). We also added measurements from the literature on proportions of belemnite rostra and phragmocones (mostly from Schlegelmilch, 1998). We took length and width measurements of the main hard parts such as rostrum, phragmocone and proostracum (Fig. 4). For the phragmocone, we also measured the apical angle since it is relevant to reconstruct the length or width of incomplete specimens. For the soft parts, we limited measurements to length. We took length measurements of the head (from the end of the proostracum to the arm bases) and arm lengths. It must be taken into account that the soft part and proostracum measurements contain uncertainties in the case of belemnites, because some show indications for predation (Klug et al., 2021) and some others likely were embedded obliquely (Fig. 1), which led to some telescoping and flattening in strongly compacted sediments such as the bituminous mudstones from Holzmaden, Germany (Reisdorf et al., 2012; Schweigert, 1999, 2018).

**Table 1:** Measurements of various belemnites (in mm)

	Rostrum			Phragmocone			Proostr	
Species	Lr	Wr	CLr	α	Lphr	Wphr	Lpro	Wpro
*M. suevica*			130.9	21	436.2	120	483.2	
*M. suevica*	290	36					181.3	
*M. suevica*	383.7	39.5			295.7		239.8	
*M. suevica*	510		190	22	393.1		318.8	
*M. elliptica*	480	48		19				
*M. elliptica*	175	25	62	20				
*M. elliptica*	700		130.9	21	436.2	153.8	483.2	
*M. elliptica*	800	100	170.1		567.1	200	567.1	
*M.* sp.	160	22		20				
*A. speciosus*								
*A. speciosus*					40		65	
*A. speciosus*	30.2	11.6		34.8	97.5	25.5	150.9	
*Acro. conoideus*	90	18		25				
*Acro. subgracilis*	85	9		27				
*Bel. baculiformis*	130	14		24				
*Belem. antiquus*					72		83	
*Ch. wunnenbergi*					90		115	
*H. hastatus*	140	12.3			98.2	22.1	86	
*H. hastatus*	70	5.9	23.8	11	65.3	13.1		
*H. hastatus*	141.4	12.9	27.9	20	92.9	21.4	102.9	34.3
*H. semisulcatus*	116	11		18				
*Meso. beneckei*	168.4	26.3		22				
*Meso.* cf. *rhenana*	130	28		25				
*N. werneri*	81	11		28				
*Neo. forthensis*	46	5		24	34	14	37.7	
*P. carinata*	111	12		21				
*P. bisulcata*	150	21		25				
*P. bisulcata*	104.8	19.4	17.5	25	58.2		64.5	
*P. bisulcata*	114.3	20	23.2	25	77.4		85.7	
*S. zellensis*					45		75	

Sources, specimen numbers and references are provided in Table 2

*M *Megateuthis; *A *Acanthoteuthis; *Acro *Acrocoelites; *Bel *Belemnopsis; *Belem *Belemnoteuthis; *C*_h_ Chondroteuthis; *H *Hibolithes; *Meso *Mesoteuthis; *N *Nannobelus*; Neo *Neoclavibelus; *P *Passaloteuthis; *S *Sueviteuthis; *L*_r_ rostrum length; *W*_r_ rostrum width; *C*_Lr_ rostrum cavum length; *α* phragmocone apical angle; *L*_phr_ phragmocone length; *W*_phr_ phragmocone width; *L*_pro_ proostr length; *W*_pro_ proostracum width

**Table 2:** Ratios and references of various belemnites

Species	Lr/Lphr	CLr/Lphr	La/Lb	Lpro/Lb	Lm/Lr	CLr/Lr	Source
*M. suevica*							PIMUZ 17125
*M. suevica*							PIMUZ 39843
*M. suevica*							Schlegelmilch, 1998: pl. 14, Fig. 5
*M. suevica*		0.48				0.37	Zieten 1831: pl. 19 Fig. 1 Schlegelmilch, 1998: p. 75
*M. elliptica*							PIMUZ 17652
*M. elliptica*						0.35	PIMUZ 21590
*M. elliptica*	1.6	0.3			1.81	0.19	Winkler coll
*M. elliptica*	1.41	0.3			2.21	0.21	Schlegelmilch, 1998: p. 25
*M.* sp.							PIMUZ 21590
*A. speciosus*							Klug et al., 2016, fin specimen
*A. speciosus*							Klug et al., 2016: Fig. 1
*A. speciosus*	0.31						Fuchs, 2015: Fig. 455e
*Acro. conoideus*							Schlegelmilch, 1998: pl. 6, Fig. 2
*Acro. subgracilis*							Schlegelmilch, 1998: pl. 6, Fig. 2
*Bel. baculiformis*							Schlegelmilch, 1998: pl. 17, Fig. 1
*Belem. antiquus*			0.31	0.32			Klug et al., 2023; NHMUK 25966
*Ch. wunnenbergi*			0.2	0.38			Klug et al., 2023, BGR MA 13436
*H. hastatus*	1.43				2.05		Fuchs, 2015: Fig. 455a
*H. hastatus*	1.07	0.36				0.34	Fuchs, 2015: Fig. 455b
*H. hastatus*	1.52	0.3			2.2	0.2	Schlegelmilch, 1998: pl. A, Fig. 3a
*H. semisulcatus*							PIMUZ 27195
*Meso. beneckei*							Schlegelmilch, 1998: pl. 14, Fig. 1
*Meso. cf. rhenana*							Schlegelmilch, 1998: pl. 11, Fig. 8
*N. werneri*							Schlegelmilch, 1998: pl. 1, Fig. 13
*Neo. forthensis*							Schlegelmilch, 1998: pl. 12, Fig. 2
*P. carinata*							Schlegelmilch, 1998: pl. 3, Fig. 2, 3
*P. bisulcata*							Schlegelmilch, 1998: pl. 2, Fig. 6, 7
*P. bisulcata*	1.8	0.3	0.36	0.18	1.92	0.167	Schlegelmilch, 1998: pl. A, Fig. 1
*P. bisulcata*	1.48	0.3			2.25	0.2	Schlegelmilch, 1998: pl. A, Fig. 2
*S. zellensis*			0.26	0.39			Klug et al., 2023 GPIT Ce 1564/2,6/PV-67025

*M *Megateuthis; *A *Acanthoteuthis; *Acro *Acrocoelites; *Bel Belemnopsis; Belem *Belemnoteuthis; *C*_h_ Chondroteuthis;* H *Hibolithes;* Meso *Mesoteuthis;* N *Nannobelus;* Neo *Neoclavibelus;* P *Passaloteuthis;* S *Sueviteuthis; *L*_r_ rostrum length; *W*_r_ rostrum width; *C*_Lr_ rostrum cavum length; *α* phragmocone apical angle; *L*_phr_ phragmocone length; *W*_phr_ phragmocone width; *L*_pro_ proostracum length; *W*_pro_ proostracum width; *L*_h_ head length; *L*_a_ arm length; *L*_m_ mantle length; *L*_b_ body length

The Slenderness-Indices (height of rostrum divided by rostrum solidum length) after Schlegelmilch (1998) are given to provide accurate relationships between rostrum diameter and length. In doing so, it is important to note that those indices concern the rostrum length, which is in our case equivalent with the rostrum solidum plus epirostrum length without the rostrum cavum length.

**Fig. 5:**
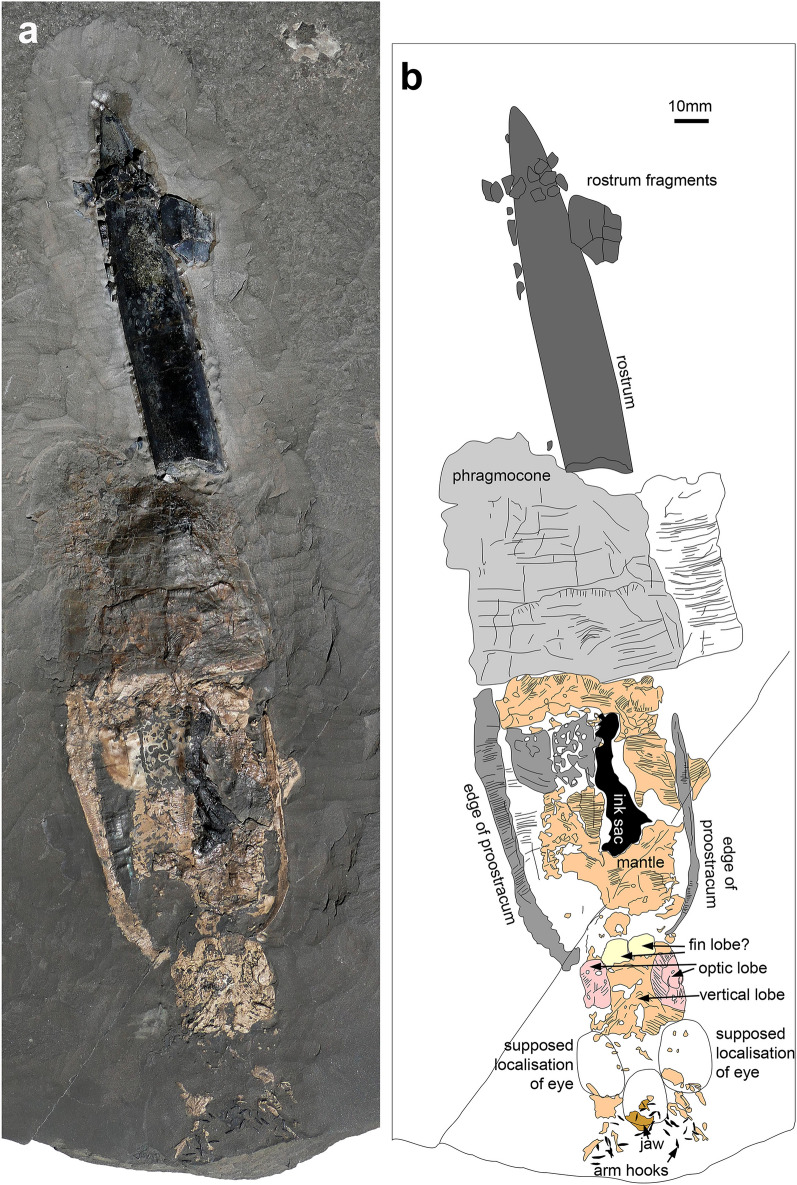
*Passaloteuthis bisulcata*, SMNS 70600, from the early Toarcian, Tenuicostatum Zone, Semicelatum Subzone, Ohmden, Germany. This specimen shows the proostracum and head region particularly well. **a** photo. **b** drawing with anatomical interpretation. Both are shown at the same scale, scale bar in **b**

**Fig. 6:**
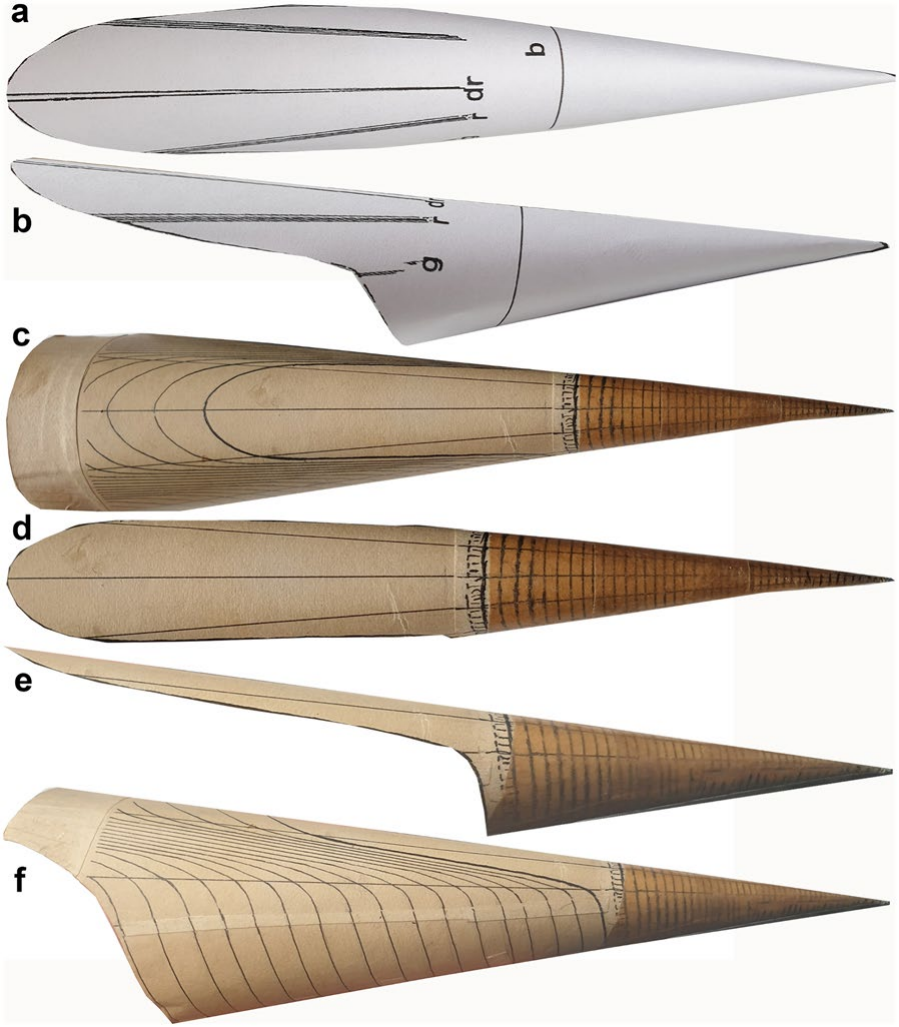
Photos of paper models of phragmocone and proostracum of *Megateuthis*. **a**, **b**, dorsal and lateral view of a model using a drawing from Doguzhaeva & Mutvei (2002: Fig. 1b). **c** to **f**, cropped (**d**, **e**) and uncropped version of a paper model made by Adolf Naef

**Fig. 7:**
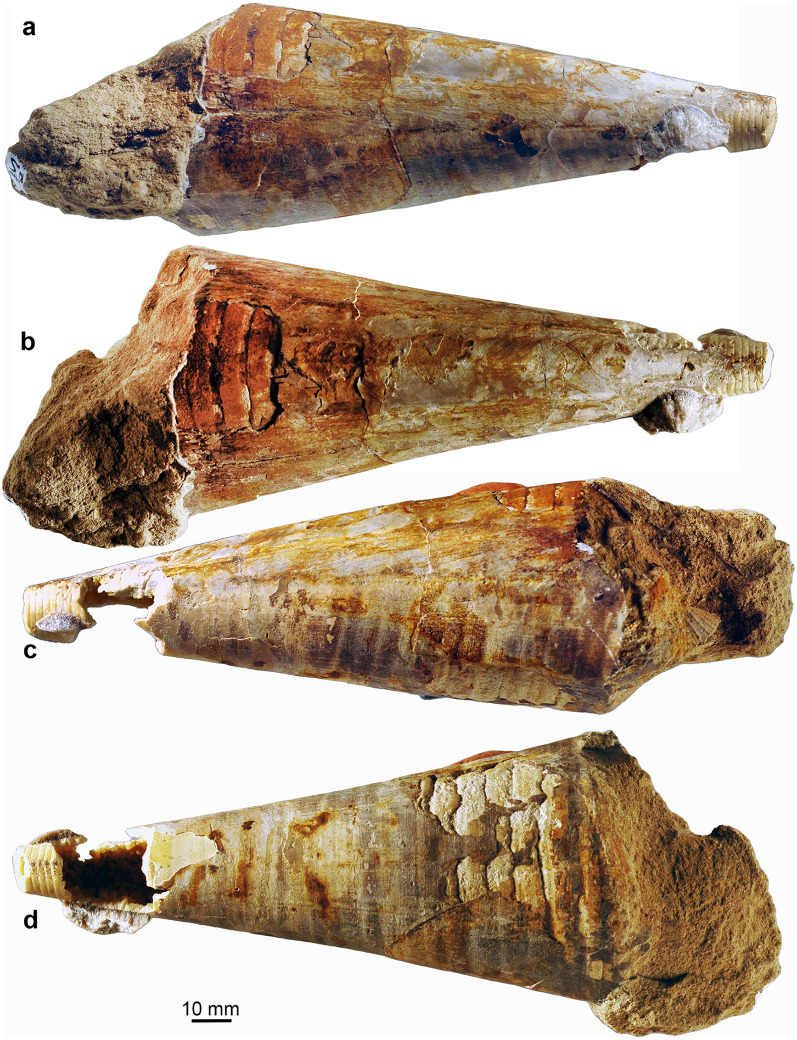
Photos of the phragmocone of *Megateuthis elliptica*, MNHNL BM567, Bajocian of Rumelange, Luxembourg. This specimen displays growth lines of the proostracum (corresponding line drawings in Fig. 7). **a** lateral; **b** dorsal; **c** lateral; **d** ventral views

**Fig. 8:**
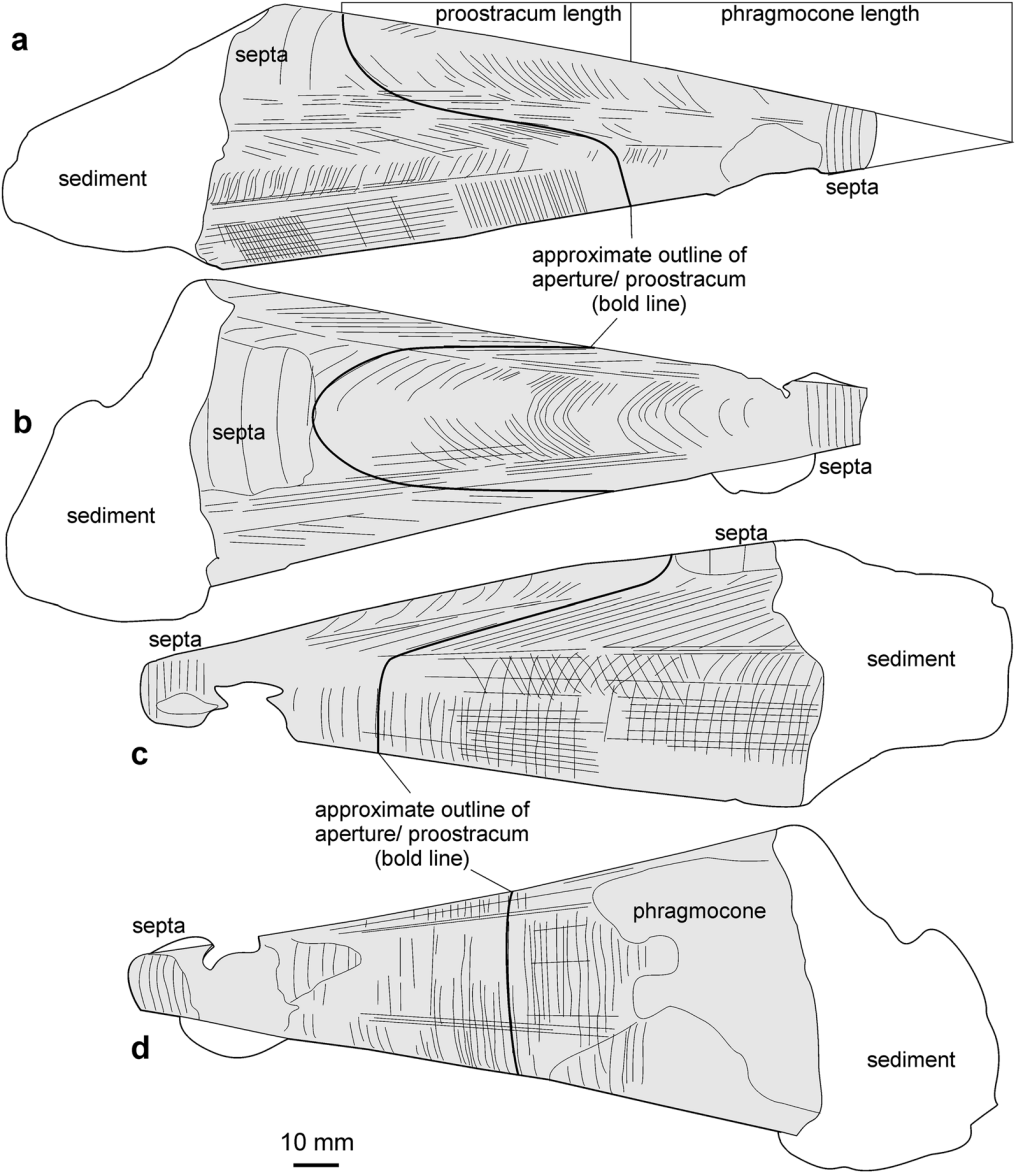
Drawings of the phragmocone of *Megateuthis elliptica*, MNHNL BM567, Bajocian of Rumelange, Luxembourg. This specimen displays growth lines of the proostracum (corresponding photos in Fig. 6). **a** lateral; **b** dorsal; **c** lateral; **d** ventral views

**Fig. 9:**
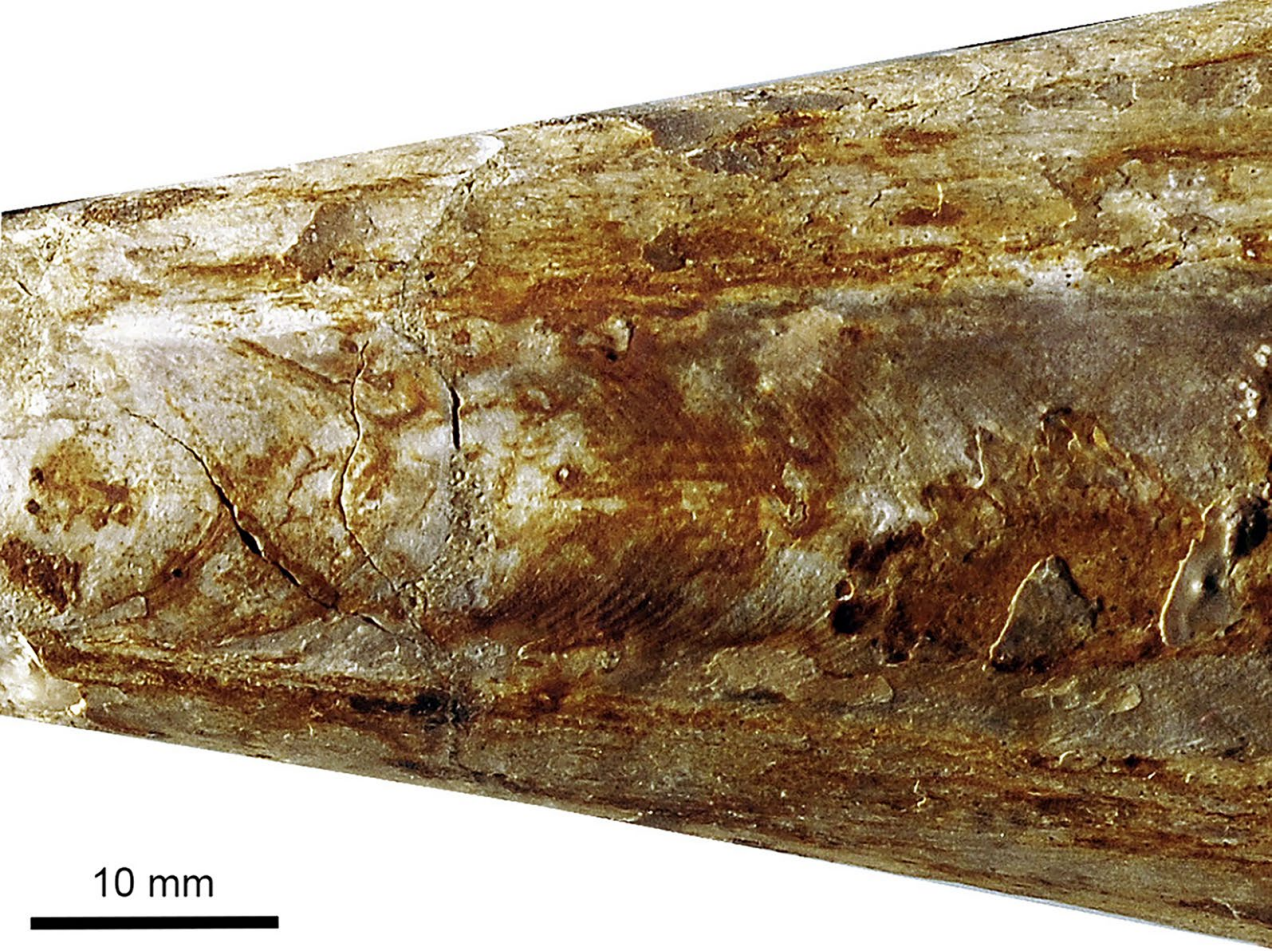
Detail of the phragmocone of *Megateuthis elliptica*, MNHNL BM567, Bajocian of Rumelange, Luxembourg, showing the growth lines on the former proostracum

A key question is also that for proostracum length, since it is only very rarely and poorly preserved in belemnites (Fig. 5), and still quite rarely in belemnotheutids (measurements and references in Tables 1 and 2). For the proostracum of *Megateuthis*, only Doguzhaeva et al. (2002) documented the course of the growth lines on the phragmocone of *Megateuthis* from Novaya Zemlya, corresponding to earlier ontogenetic stages of the proostracum. For visualisation, we printed their Fig. 1 on paper, combined them with a corresponding triangle for the phragmocone surface, cut out the phragmocone-proostracum outline and then rolled and glued it as a model for these hard parts (Fig. 6). We compared our paper-model with a historical one that was produced by A. Naef (Fig. 6c–f). For the phragmocone, we used an apical angle of 20°, which corresponds to the angle in several undistorted specimens of *Megateuthis*. Additionally, we examined a phragmocone of *Megateuthis elliptica*, BM567, Bajocian of Rumelange, Luxembourg, with replacement shell (Figs. 7, 8, 9). The reconstructed former apertures and proostracum length proportions correspond well to Naef’s and Doguzhaeva’s models.

Since the mantle ended likely near the anterior edge of the proostracum, we use the term ‘mantle length’, which is widely used by neontologists as a measure of body size (Jereb & Roper, 2005; Roper & Voss, 1983), for the length of the hard parts from the apex of the rostrum to the anterior end of the proostracum. While it is simple to obtain values for maximum rostrum and phragmocone lengths, it is less trivial to establish the degree of overlap of the rostrum with the phragmocone, i.e. the length of the rostrum cavum. For this purpose, we used both sectioned specimens and others preserving much of the phragmocone still inside the rostrum (Figs. 2, 3).

For the reconstruction of soft part proportions, we had to rely on the few known soft part belemnites (examples in Figs. 1, 5). Although belemnite rostra and phragmocones are regionally very abundant in many countries, belemnites preserving their mineralized soft parts and remains of arm crowns are still limited to southern Germany (Fuchs & Hoffmann, 2017; Klug et al., 2010, 2020, 2021; Reitner & Urlichs, 1983; Riegraf & Hauff, 1983; Schlegelmilch, 1998; this study). We also measured some belemnotheutids with complete bodies to improve our database (Table 1, 2 and references therein). In particular, we measured the lengths from the anterior edge of the proostracum to the arm bases (head length) and the arm length from the bases. Because of the absence of internal hard parts in the arms, their length may vary; accordingly, head and arm crown are usually not included in the neontological literature when size data are provided (Jereb & Roper, 2005). It must be pointed out that soft parts and the according proportions of early growth stages of belemnites are unknown.

### Proportions of the mineralized hard parts

The longest rostra, of which we obtained photos, are about 70 cm long (Fig. 2f). According to the materials we examined in various European museum collections and the measurements of Schlegelmilch (1998), the rostrum of *Megateuthis elliptica* grew longer (Slenderness Index: 0.10 to 0.11) but did not reach the same diameter as *M. suevica* (formerly *M. gigantea*) (Slenderness Index: 0.15 to 0.37). These two species are widespread and therefore, their sizes are discussed in detail here.

Schlegelmilch (1998: p. 75) suggested that the largest rostra might have attained 80 cm, but without providing the source of that measurement. Following our reasoning above, we assume that these long rostra belong to *M. elliptica*. Hence, we use these two alternative values (70 and 80 cm) for the length of the complete rostrum including rostrum solidum (Müller-Stoll, 1936: the part from the tip of the rostrum to the initial chamber/ protoconch) and rostrum cavum (the part of the rostrum surrounding the apical portion of the phragmocone from the initial chamber/ protoconch to the anterior edge of the rostrum; Fig. 4) of *M. elliptica*. The greatest rostrum length of *M. suevica* was provided by Zieten (1831: pl. 19 Fig. 1) and Schlegelmilch (1998: p. 75) as being 51 cm.

Epirostra are formed near adulthood (Arkhipkin et al., 2015; Bandel & Späth, 1988; Stevens et al., 2022); this part is often hollow, and the proportion of the total rostrum length varies between 25% in *M. elliptica* (Schlegelmilch, 1998: p. 76), 36% in *M. suevica* (Bandel & Späth, 1988: p. 253), and nearly 80% in *Acrocoelites gracilis* (= “*Belemnites acuarius tubularis*” in Bandel & Späth, 1988: p. 252). Some specimens of *M. suevica* display slightly offset epirostra, which have a lower apical angle than the orthorostra (Fig. 2b). Among the rostra we examined, however, the rostra with distinct epirostrum were not the largest, because they mostly belong to the thicker but shorter *M. suevica*.

Since it is nearly perfectly conical, the maximum length L_phr_ of the phragmocone can be calculated (at least approximately, because sometimes, the apical angle changes slightly through ontogeny; Bandel & Späth, 1988: pp. 252, 253) with the knowledge of its apical angle α (around 20°; see Tab. 1–3) and the largest diameter D_phr_ of the largest phragmocone fragment:

$${{\text{L}}}_{{\text{phr}}}=\frac{{{\text{D}}}_{{\text{phr}}}}{2\times {\text{tan}}\left(\frac{\alpha }{2}\right)}=\frac{20 cm}{2\times {\text{tan}}\left(\frac{20^\circ }{2}\right)}=56.71 cm$$

Some of the largest phragmocones available to us, which were found in Luxembourg, are shown in Fig. 3. Their terminal diameters are 14 cm in a specimen with rostrum and 15.4 cm in an isolated phragmocone fragment from Ringsheim. Schlegelmilch (1998) suggested 20 cm as maximal phragmocone diameter based on isolated chambers, but he did not mention a repository. Using a phragmocone diameter of 15 cm yields a length of 42.5 cm.

**Tab 3:** Calculated dimensions of *Megateuthis* body parts, based on different model assumptions

Species	S98-N22	Kea-N22	S98-D02	Kea-D02	Z31-N22	Z31-D02
	*M. elliptica*	*M. elliptica*	*M. elliptica*	*M. elliptica*	*M. suevica*	*M. suevica*
Dphr	20 cm	15 cm	20 cm	15 cm	15 cm	15 cm
Lr	80 cm	70 cm	80 cm	70 cm	51 cm	51 cm
alpha	20°	20°	20°	20°	20°	20°
Ppp	1.00	1.00	0.78	0.78	1.00	0.78
Prc	0.3	0.3	0.3	0.3	0.3	0.3
Ph	0.12	0.12	0.12	0.12	0.12	0.12
Pa	0.64	0.64	0.64	0.64	0.64	0.64
Pr	0.45	0.49	0.49	0.53	0.41	0.45
Lphr	56.7 cm	42.5 cm	56.7 cm	42.5 cm	42.5 cm	42.5 cm
Lrc	17.0 cm	12.8 cm	17.0 cm	12.8 cm	12.8 cm	12.8 cm
Lpo	56.7 cm	42.5 cm	44.2 cm	33.2 cm	42.5 cm	33.2 cm
Lm	176.4 cm	142.3 cm	163.9 cm	133.0 cm	123.3 cm	114.0 cm
Lh	21.2 cm	17.1 cm	19.7 cm	16.0 cm	14.8 cm	13.7 cm
La	112.9 cm	91.1 cm	104.9 cm	85.1 cm	78.9 cm	72.9 cm
Total	310.5 cm	250.5 cm	288.5 cm	234.0 cm	217.0 cm	200.6 cm

Parameters in bold are calculated, see text for details. Models are labled as follows: S98 = maximum values given by Schlegelmilch (1998) for rostrum length and phragmocone diameter; Con = more conservative estimates found in this study. Z31 = specimen of Zieten (1831); N22 = Proostracum proportion given by Naef (1922); D02 = Proostracum proportion given by Doguzhaeva et al. (2002)

*D*_phr_ phragmocone diameter; *L*_r_ rostrum length; *alpha* apical angle of phragmocone; *P*_pp_ Proportion between

proostracum and phragmocone; *P*_rc_ Proportion of rostrum cavum compared to phragmocone; *Ph* proportion of head compared to mantle length; Pa = proportion of arms compared to mantle length; *Pr* proportion of rostrum compared to complete hard parts; *L*_m_ mantle length; *L*_phr_ phragmocone length; *L*_rc_ rostrum cavum length; *L*_po_ Proostracum length; *L*_h_ head length; *L*_a_ arm length

Depending on the model used, we obtained varying proportions of the main body parts along the longitudinal axis. Here, we assess the maximum length of *Megateuthis* and thus *M. elliptica*. A comparison to *M. suevica* is provided later. The proportions used for *M. elliptica* are as follows (see Tables 2, 3):

1. Ratio of rostrum, total length, to length of internal hard parts including proostracum (mantle length): 0.47 with a range from 0.44 to 0.52 in *Passaloteuthis* preserving soft-parts.

2. Ratio of rostrum cavum to total phragmocone length is around 0.3. Using this ratio and the above calculated values for phragmocone length, we calculated a length of 17.0 cm for the rostrum cavum at a phragmocone diameter of 20 cm, and 12.8 cm at 15 cm phragmocone diameter.

3. Phragmocone length is similar to proostracum length. In Naef’s model (Fig. 6c to f), the ratio is 1:1. In our paper model (Fig. 6a, b) using the proostracum shape given by Doguzhaeva et al. (2002), combined with an apical angle of the phragmocone of 20°, the proostracum is 78% of the phragmocone length. Accordingly, the proostracum would reach between 44.2 and 56.7 cm at a phragmocone diameter of 20 cm and between 33.2 and 42.5 cm at 15 cm phragmocone diameter.

4. Hard part length (total shell length) of belemnites corresponds to mantle length and can be determined by subtracting rostrum cavum length from the rostrum length (= rostrum solidum and epirostrum) and add phragmocone and proostracum lengths. At 20 cm phragmocone diameter and 80 cm rostrum length (the values proposed by Schlegelmilch, 1998), this would amount to a total shell length and approximate mantle length in *M. elliptica* of 176.4 cm and 163.9 cm at 78% proostracum length. Using the better documented values of 15 cm for phragmocone diameter and 70 cm for rostrum length, we obtain a mantle length of 142.3 cm as a more conservative estimate (133 cm at 78% proostracum length). For *M. suevica,* we obtained a mantle length between 114.0 cm and 123.3 cm at a rostrum length of 51 cm and a phragmocone diameter of 15 cm.

Accordingly, mantle length (Lm) can be summarised in the following formula, where D_phr_ = max. phragmocone diameter, α = phragmocone apical angle, P_pp_ = proportion between proostracum and phragmocone, P_rc_ = proportion between rostrum cavum and phragmocone, and L_r_ = total rostrum length:

$${\text{L}_m}=\frac{{{\text{D}}}_{{\text{phr}}}}{2\times {\text{tan}}\left(\frac{\alpha }{2}\right)}\times \left({1+P}_{pp}-{P}_{rc}\right)+{L}_{r}$$

### Soft part proportions

The more or less complete belemnite animal is known only from a few specimens of the Toarcian *Passaloteuthis bisulcata* and the Kimmeridgian *Hibolithes semisulcatus* (Klug et al., 2010, 2021; Riegraf & Hauff, 1983; Schlegelmilch, 1998). According to a recent phylogenetic study, *Passaloteuthis* is more or less closely related to *Megateuthis* (Stevens et al., 2023), so the anatomy of the two taxa can be expected to be similar. In *P. bisulcata* (Figs. 1, 5), the head region is c. 2.4 cm long, the arms attain about 12.9 cm at a mantle length of 20.2 cm and a whole-body length with arms of 35.5 cm. Accordingly, head length makes up 6.8% of full body length and 12% of mantle length. Concerning arm lengths, these would measure about 36% of full body length and 64% of mantle length.

Applying these values to *Megateuthis elliptica*, we obtain a head length between 21.2 cm for 20 cm phragmocone width and 1:1 phragmocone to proostracum ratio, and 16 cm for 15 cm phragmocone diameter and 78% proostracum length. Correspondingly, we can calculate arm lengths between 112.9 cm and 85.1 cm. If we apply the same to *M. suevica*, we obtain a head length of up to 14.8 cm and an arm length of up to 78.9 cm.

## Results and discussion

### Maximum body size

**Fig. 10:**
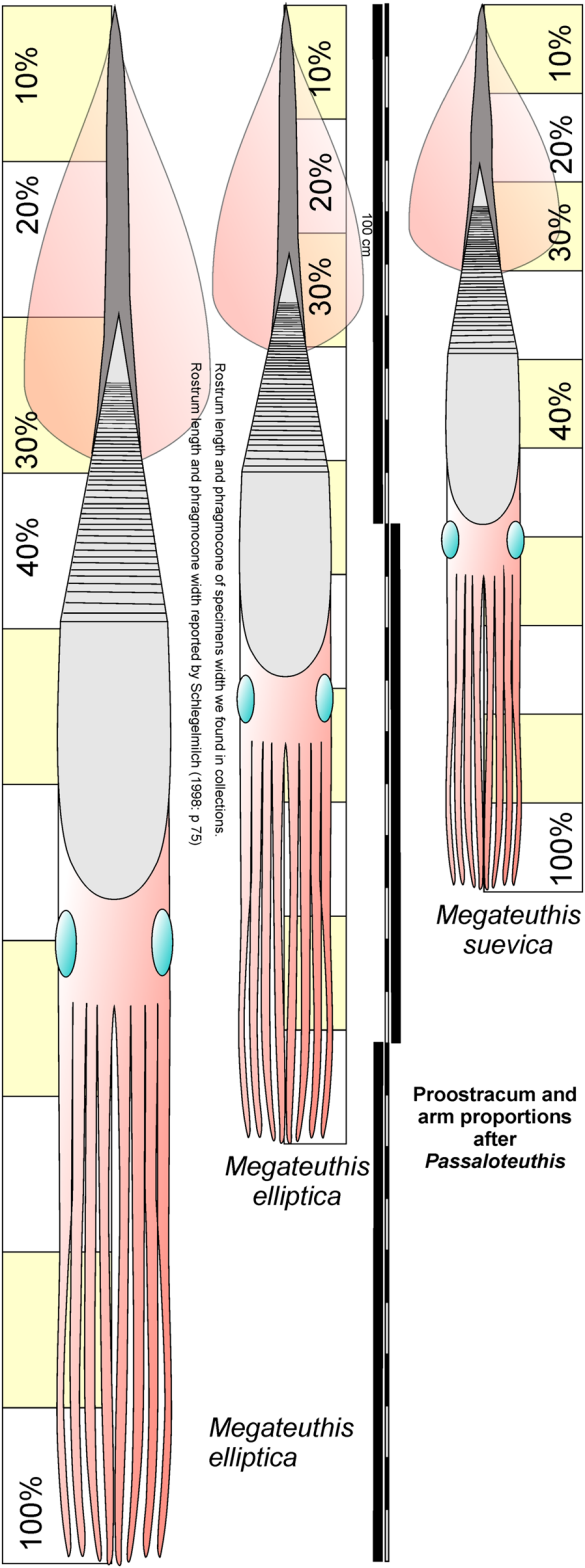
Reconstructions of *Megateuthis elliptica* combining two alternative assumptions for maximum rostrum length and maximum phragmocone diameter and of *M. suevica* using proportions of the soft tissue-specimens of *Passaloteuthis* (Figs. 1, 5)

Using the values given above and the estimates by Schlegelmilch (1998), the complete animal of *Megateuthis elliptica* including head and arms would have reached between 234 and 310.5 cm depending on the maximum phragmocone diameter (15 vs. 20 cm), proostracum proportion (0.78 vs. 1.0 of phragmocone length) and rostrum length (70 vs. 80 cm) as explained above (Fig. 10). It was likely the longest cephalopod of the Jurassic, although with respect to body weight, there may have been heavier ammonoids (we also do not know the arm length of ammonoids), whose coiled conchs reached possibly up to 1.5 m in diameter in the Bajocian and the Kimmeridgian (Dietl & Hugger, 1979; Stevens, 1988), or nautilids with conchs up to 61 cm diameter (Weis et al., 2023) or even 77 cm (Grulke, 2016). *M. suevica* was shorter with body lengths of up to 217 cm, but it was probably a bit more robust and, in the end, the body mass of the largest individuals of these two species might have been similar.

At a maximum estimated mantle length of at least 1.33 m and possibly up to 1.76 m, *Megateuthis* got close to the giant squid *Architeuthis dux* (mantle length of 2.4 m; Landman et al., 2004; see for even larger estimates of *Architeuthis* up to 2.69 m mantle length in Paxton, 2016), *Mesonychoteuthis hamiltoni* (mantle length of 3 m; Rosa & Seibel, 2010; Rosa et al., 2017), *Onykia robusta* (mantle length of 3 m; Bolstad, 2008), *Megalocranchia maxima* (mantle length of 2 m; Kubodera & Horikawa, 2005), *Taningia danae* (mantle length of 1.7 m; Roper & Jereb, 2010) and *Dosidicus gigas* (mantle length of 1.5 m; Wormuth, 1976). Thus, adult *Megateuthis* would have ranked among the ten largest cephalopod species today.

These extreme values are not in contrast with the assumed relatively short life span of one to two years as calculated based on growth increments by Dunca et al. (1996). For example, the Recent giant Pacific Octopus *Enteroctopus dofleini* reaches an arm span of up to 6 m in three to six years (Cosgrove, 2009). Estimates of lifespans between three to six years have also been suggested for giant squids based on modelling (Grist & Jackson, 2007; Perales-Raya et al., 2020). Landman et al. (2004) reported a relatively long lifespan of 14 years or less for three female specimens of the giant squid *A. dux* captured off Tasmania, Australia, based on Δ^14^C analyses of statoliths.

### Shape and position of fins

So far, the fin remains documented by Klug et al. (2016) for two Late Jurassic *Acanthoteuthis speciosa* specimens represent the only records for belemnoids. In one case, they are rather short and terminal, attached to the very thin rostrum, while in the other specimen, they are proportionally almost twice as long. In the absence of fossilized fins in belemnitids proper, we are limited to list plausible scenarios. It appears likely that the fins had a subapical to apical position and were attached to the rostrum (Bandel & Späth, 1988; Monks et al., 1996; Naef, 1922). It has been suggested that the fin cartilage was attached to the lateral furrows (Fuchs et al., 2016), but in *Megateuthis*, these can split into four at the apex (Schlegelmilch, 1998). This raises the question whether it had two or four fins like the Jurassic octobrachian *Trachyteuthis* (ref. Fuchs & Schultze, 2008: Fig. 6A) and *Plesioteuthis* (Klug et al., 2015b) or juveniles of the Recent *Vampyroteuthis* (Pickford, 1949). In some living decabrachians, four fins remain in adult animals, e.g., *Grimalditeuthis* (Hoving et al., 2013; Joubin, 1898). However, these coleoids are rather distantly related and the four apical folds form only late in ontogeny (Schlegelmilch, 1998), letting us assume that there were only two fins throughout life. Naef (1922: figs. 74–76) placed the fins in belemnites like *Hibolithes* quite anterior on the rostrum while Klug et al., (2010: Fig. 8) put it in a terminal position where the fins attach over more or less the entire rostrum length.

This leads to the question of the main function of the rostrum: Did it help to balance the belemnite body in a horizontal position for reduced drag during rapid swimming (Jenny et al., 2019; Monks et al., 1996) or did it serve primarily for the attachment of the fins (discussion in Hoffmann & Stevens, 2020)? In the latter case, we would have to assume that the fin cartilage was really attached over most of the rostrum length. In any case, fossil belemnites preserving fin remains are needed to test these hypotheses.

### Was there a Bajocian marine animal gigantism?

Neither was *Argovisaurus* (Miedema et al., 2024) the largest ichthyosaur of the Jurassic, nor were the ammonites the largest of all times (Stevens, 1988). Nevertheless, the middle European region is remarkable in the co-occurrence of several big animal species. With a reconstructed skull length of 1.38 m and a body length exceeding 6 m, *Argovisaurus* (Miedema et al., 2024) was on the tall side, particularly for post-Toarcian ichthyopterygians. Concerning pliosaurids, they likely became marine apex predators around that time. Benson et al. (2013), Sachs et al., (2019, 2023) and Madzia et al. (2022) portrayed large pliosaurs with up to two-meter-long skulls and document their rise to the top of the marine food web with an important increase in body size in the Bajocian.

Concerning invertebrates, bivalve faunas can be incredibly rich. Hallam (1976, 1977) documented the rise in bivalve diversity in the Bajocian. There are also several large species such as a *Modiolus giganteus* with a shell length of 15 cm (Schweigert, 2011: p. 198). Further, the terebratulid brachiopods *Gigantothyris* and *Morrisithyris* are regionally common and grew to lengths exceeding 5 cm (e.g., Alméras et al., 2007: Fig. 5.5).

However, the sizes of Bajocian cephalopods stand out. In his ammonoid size study, Stevens (1988) cited Dietl and Hugger (1979), who had claimed to have found a poorly preserved *Lytoceras* that measured 1.5 m in diameter, 24 cm smaller than the current world record concerning ammonite diameter (Stevens, 1988 and references therein). However, they neither saved the specimen nor provided a field photo to document its size, casting some doubt on this interpretation. As mentioned by Miedema et al., (2024: Fig. 2C), a lytoceratid phragmocone was found with a large ichthyosaur and a *Megateuthis* (PIMUZ 39843) with estimated (mantle) length of 55 cm (Fig. 2a). This lytoceratid phragmocone has a diameter of 41 cm and likely had a diameter of around 70 cm including the body-chamber. Similarly, Weis et al. (2023) described huge nautilids from the Middle Jurassic. They introduced the species *Cenoceras rumelangense*, which reaches at least 61 cm in diameter. Nautilids of similar size are known from Germany (53.5 cm according to Weis et al., 2023) and England (77 cm according to Grulke, 2016: p. 138).

**Fig. 11:**
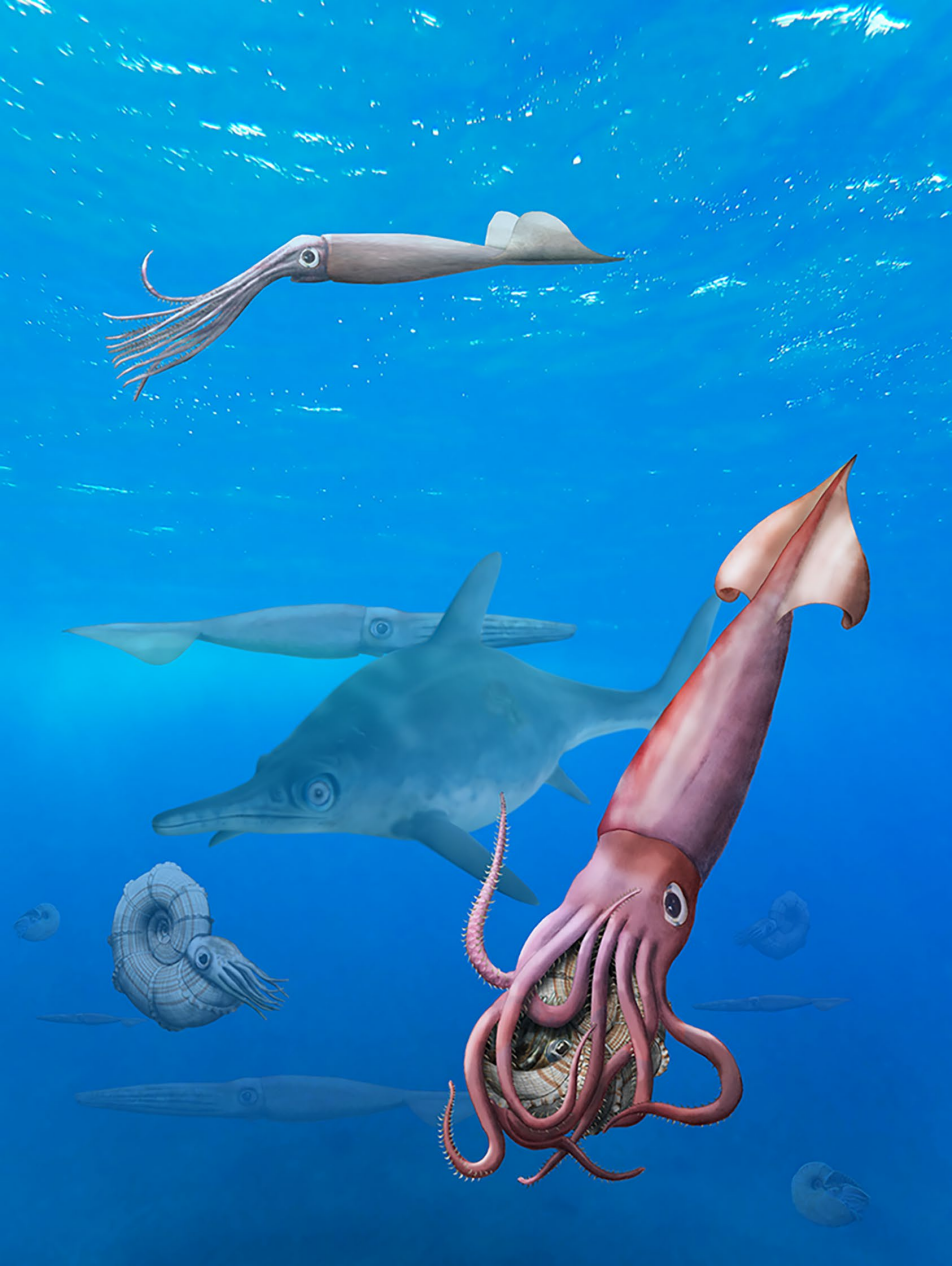
A scene from the Germanic Basin during the Bajocian (e.g. in Germany, Luxembourg or Switzerland) with *Megateuthis suevica* catching a lytoceratid, a five-meter-long ophthalmosaurid like *Argovisaurus,* and nautilids in the background. These species co-existed in central Europe, but there is no direct evidence for their actual interactions

Klug et al. (2015a) investigated the temporal and spatial controls on Paleozoic marine invertebrate gigantism and could demonstrate relationships with increased diversity as well as environmental factors like oxygenation, temperature and sea level. Increased diversity, disparity or preservation are unlikely to explain this pattern in Bajocian belemnites as the diversity, disparity and preservation of Bajocian belemnites seem eclipsed by the Toarcian (Dera et al., 2016), but further analyses comparable to those in the Lower Jurassic (De Baets et al., 2021; Neige et al., 2021; Rita et al., 2021) are necessary to further corroborate these patterns. Meer et al. (2022) studied sea-level changes in the Phanerozoic. In their overview, they show that a Middle Jurassic sea-level rise was found by several authors. Following this and the reasoning by Klug et al. (2015a), the above-mentioned groups maybe simply have encountered favorable conditions. Remarkably, some of the huge cephalopods mentioned above including *Megateuthis* are known from more shallow marine facies than normally expected. However, Wiggan et al. (2018) also highlighted major changes in productivity driven by a more humid climate as well as ocean circulation linked with a major radiation in the pelagic realm during the Bajocian postulating a possible underlying ecological driver linked with the Mesozoic Marine Revolution (Vermeij, 1977). It is also conceivable that the simultaneous (co)evolution of large forms in vertebrate predators and invertebrate prey is best explained by biotic factors (Red Queen and other related models: Benton, 2009; van Valen, 1973; Voje et al., 2015) such as increased competition for resources or escalation of predator–prey relationships (Fig. 11). In any case, this phenomenon would deserve a study on its own including data from various groups finely sampled over a longer stratigraphic interval, thoroughly evaluated statistically.

### Was there a sexual dimorphism?

There is no hard evidence for sexual dimorphism in belemnites yet. Concerning the two large forms *Megateuthis suevica* and *M. elliptica*, their widely overlapping occurrence and their similar morphologies are remarkable. Such species pairs evoke the question for sexual dimorphism, which has been discussed for belemnites a few times, in particular referring to the presence and absence of large hooks (so-called mega-onychites) in *Passaloteuthis* (Engeser & Clarke, 1988; Engeser, 1987; Hoffmann et al., 2017; Klug et al., 2021; Reitner & Urlichs, 1983; Riegraf & Hauff, 1983; Schlegelmilch, 1998; Stevens, 2010). These large hooks (Fig. 1c), however, are almost exclusively known from the Jurassic (but see Bonde et al., 2008).

## Conclusions

We document some of the largest remains of the largest known belemnite genus, the Bajocian *Megateuthis*. At rostrum lengths of 70 cm and phragmocone diameters of at least 15 cm, mantle length may have reached around 1.33 or even 1.76 m, which would rank it among the ten largest modern coleoids. When using the proportions of the few soft body belemnites (*Passaloteuthis* from the Toarcian), we get full body lengths including arms of up to 2.17 m in the shorter and more robust *M. suevica* and possibly up to 3.11 m (2.34 m using more conservative estimates for individual hard parts) in the more slender but longer *M. elliptica*. While it is parsimonious to assume proportions and anatomy similar to the better-known Toarcian *Passaloteuthis*, we still lack reliable information about shape, size and position of the fins in this clade. The remarkable size (co)evolution of both Bajocian marine reptile predators and molluscan prey species like cephalopods is tentatively explained by favourable abiotic and biotic factors.

## Data Availability

All data and measurements are either from the literature as referenced or from museum specimens. The measurements are provided in the tables. Some of the referenced materials are illustrated.
